# Corneal viscoelasticity is associated with intraocular pressure under physiological baseline: insights from the rheological properties of corneal lenticules

**DOI:** 10.3389/fbioe.2025.1694568

**Published:** 2025-11-11

**Authors:** Xiaonan Yang, Qi Ren, Xiangbin Kong, Peng Su, Chang Liu, Quan Liu, Pengxia Wan

**Affiliations:** 1 Department of Ophthalmology, The First Affiliated Hospital, Sun Yat-sen University, Guangzhou, China; 2 Department of Ophthalmology, The Second People’s Hospital of Foshan, Foshan, China; 3 State Key Laboratory of Ophthalmology, Zhongshan Ophthalmic Center, Sun Yat-sen University, Guangdong Provincial Key Laboratory of Ophthalmology and Vision Science, Guangdong Provincial Clinical Research Center for Ocular Diseases, Guangzhou, Guangdong, China; 4 Bright Eye Hospital Group, Guangzhou, China

**Keywords:** corneal biomechanics, viscoelasticity, intraocular pressure, small incision lenticule extraction (SMILE), CorVis ST, torsional shear rheometry

## Abstract

This study aims to investigate the modulation effect of baseline intraocular pressure (IOP) on corneal viscoelastic modulus within physiological ranges. We collected 48 stromal lenticules from 26 healthy myopic patients undergoing SMILE surgery. Based on biomechanically corrected IOP (bIOP), stratifying the samples into a low-pressure group (bIOP <15 mmHg, n = 15) and a high-pressure group (bIOP ≥15 mmHg, n = 33) according to pre-operative measurements. Each fresh lenticule underwent strain-controlled torsional rheometry at 37 °C (shear strain 1%, angular frequency 0.1–100 rad s^-1^), recording storage modulus (*G′*), loss modulus (*G″*), complex viscosity (η*), and loss factor (tan *δ*), with elastic modulus (*E*) calculated from G′. In parallel, *in vivo* corneal deformation and stiffness parameters were obtained using the Corvis ST. The results showed that viscoelastic parameters increased monotonically with frequency, demonstrating solid-like behavior; in the frequency range of 10^0^–10^1.5^ rad s^-1^, *G′* and *E* were significantly higher in the high-pressure group compared to the low-pressure group (both *p* < 0.05), while the log-modulus versus log-frequency slopes showed no significant difference, indicating an upward “stiffness offset” due to elevated bIOP without altering dispersive characteristics. Corvis ST also confirmed that the high-pressure group exhibited smaller deformation amplitudes and higher stiffness parameters. Overall, even within the normal range, elevated baseline IOP results in an upward shift in corneal *E* without affecting its time-dependent properties, suggesting that corneal stromal rigidity is adaptable to the ocular pressure environment under physiological conditions.

## 1 Introduction

The cornea is the eye’s primary refractive element and a frontline protective barrier, so its biomechanics are crucial for maintaining normal vision (Meek et al., 2025). Under physiological intraocular pressure (IOP), the cornea undergoes only minute deformations, with strains typically below 1%, within which it behaves as an essentially linear viscoelastic solid (Pang, 2021). That response is governed by the architecture of the collagen fiber network, the composition of the stromal matrix, and tissue hydration. Even within the physiological pressure range, individual corneas can respond quite differently (Vinciguerra et al., 2020; Xu et al., 2021; Liu et al., 2023; Wei et al., 2023). Quantifying corneal viscoelasticity under physiological IOP is therefore fundamental to understanding structure–function coupling and disease mechanisms in disorders such as glaucoma and keratoconus.

*In vivo*, the Corvis ST uses a calibrated air puff and ultra-high-speed Scheimpflug imaging to capture dynamic corneal deformation. It yields pressure-independent indices such as biomechanically corrected IOP (bIOP) and device-derived metrics including stiffness parameter at first applanation (SPA1), stiffness parameter at highest concavity (SPHC), Ambrósio’s Relational Thickness in the horizontal direction (ARTh), Corvis Biomechanical Index (CBI), and the stress–strain index (SSI), among others (Renato et al., 2013; Roberts, 2014; Vinciguerra et al., 2016; Roberts et al., 2017; Eliasy et al., 2019; Vinciguerra et al., 2021; Zhang et al., 2021; Flockerzi et al., 2022; Miao et al., 2024). In addition, several derived parameters leverage dynamic corneal response (DCR) metrics to link biomechanics-related ocular diseases such as glaucoma—for example, the Biomechanical Glaucoma Factor (BGF) (Pillunat et al., 2019). These parameters reflect the cornea’s resistance to transient loading, complementing *ex vivo* material measurements by sampling the tissue’s performance at the organ scale under physiological conditions. Moreover, small incision lenticule extraction (SMILE) offers an opportunity to probe corneal stromal mechanics in humans. In SMILE, a femtosecond laser creates an intrastromal lenticule that is manually dissected and extracted through a small incision to correct myopia. The retrieved stromal lenticule preserves native extracellular matrix and collagen lamellae at physiologic hydration if handled promptly, enabling high-fidelity, *ex vivo* rheological testing under controlled conditions.

Quantifying corneal viscoelasticity has been approached using a range of complementary methods. Conventional rheological techniques include stress-controlled and strain-controlled oscillatory tests and steady-shear measurements. Meanwhile, strain-controlled frequency sweeps at small strains are particularly suitable for delicate collagenous tissues because they minimize structural damage while mapping storage modulus (*G′*), loss modulus (*G″*), complex viscosity (η*), and loss factor (tan *δ*) across a physiologically relevant frequency band. By limiting the applied deformation to the 0.001%–1% range, these sweeps preserve the collagen framework while probing a wide frequency band (0.1–100 rad s^-1^) that covers the characteristic rates of fixational eye movements and spontaneous retinal venous pulsations (Kim et al., 2014; Beylergil et al., 2022).

Within this context, we examined how baseline, physiological bIOP modulates corneal viscoelasticity. We hypothesized that, within the physiological IOP range, higher baseline pressure would be accompanied by an upward shift in both *G′* and *G″*, a trend that should be observable at the macroscopic (whole cornea) and microscopic (stromal layer) scales alike. We tested this by performing small-strain, strain-controlled torsional-shear frequency sweeps on fresh stromal lenticules obtained during SMILE and, in parallel, by characterizing *in vivo* corneal deformation with the Corvis ST. Establishing a quantitative link between physiological bIOP and viscoelastic modulus may refine constitutive models and inform personalized risk stratification in glaucoma, keratoconus, and pressure-related ocular disorders.

## 2 Methods

This study was approved by the Ethics Committee of the Zhongshan Ophthalmic Center (2013MEKY036) and was conducted in accordance with the Declaration of Helsinki. Written informed consent was obtained from all participants before enrollment, permitting the use of their clinical data for research.

Before surgery, each subject underwent a comprehensive ophthalmic evaluation that included slit-lamp biomicroscopy, non-contact tonometry, and anterior-segment tomography. Exclusion criteria were: 1) keratoconus or suspected keratoconus; 2) IOP <10, or >21 mmHg, normal-tension glaucoma (NTG) or suspected NTG; 3) active ocular or systemic disease; 4) prior ocular trauma or surgery; and 5) any other condition known to influence corneal biomechanics.

### 2.1 *In vivo* data

*In vivo* corneal biomechanics were assessed with the Corvis ST (Oculus, Wetzlar, Germany; software version 1.6r2187) pre-operatively and 1 day post-operatively. Only measurements labelled “OK” in the quality-specification (QS) window were included in the analysis.

### 2.2 *Ex vivo* data

SMILE was performed by a single experienced surgeon using a VisuMax femtosecond laser system (Carl Zeiss Meditec AG, Jena, Germany). After creation and manual dissection, each stromal lenticule was removed through a small incision and immediately immersed in sterile BSS Sterile Irrigating Solution (Alcon Laboratories, Inc., Fort Worth, TX, USA) at 4 °C to maintain tissue hydration.

*Ex vivo* rheological properties of the lenticules were measured on a DHR-2 rheometer (TA Instruments, USA) using a 20 mm parallel-plate geometry with a 0.9 mm gap. To prevent slippage and ensure no-slip boundary conditions, 320 grit sandpaper was glued to both loading platens (Hatami-Marbini, 2014). Prior to testing, specimens were equilibrated in BSS solution at 37 °C for 30 min. The submersion chamber of the rheometer was filled with BSS solution to maintain tissue hydration throughout measurements. Oscillatory frequency sweeps were performed at 37 °C with 1% strain and an angular frequency (*ω*) of 0.1–100 rad s^-1^. The *G′*, *G″*, η*, and tan *δ* were calculated to characterize viscoelastic behavior. All biomechanical tests were completed within 2 hours of extraction to minimize tissue degradation. The detailed rheological testing parameters are summarized in Table 1.

**TABLE 1:** Rheological testing parameters.

Parameter	Specification
Rheometer	DHR-2 (TA Instruments, USA)
Geometry	20 mm parallel plate
Gap	0.9 mm
Plate surface	320 grit sandpaper
Temperature	37 °C
Pre-equilibration	30 min in BSS at 37 °C
Test environment	BSS submersion chamber
Strain amplitude	1%
Angular frequency	0.1–100 rad s^-1^
Test mode	Oscillatory frequency sweep
Time post-extraction	<2 h

Although the cornea is anisotropic and viscoelastic, it is commonly approximated as an isotropic, linear-elastic material under small deformations. Under this assumption the elastic modulus (*E*) was derived from the storage modulus using

$$E=2G^{\prime }\left(1+\nu \right)$$

with a Poisson’s ratio (ν) of 0.40 (Ford et al., 2011).

### 2.3 Statistical analysis

All statistical analyses were performed using R (version 4.5.0). A total of 48 corneal stromal lenticule specimens from 26 healthy young myopic adults were stratified into two groups based on bIOP: the low-pressure group (bIOP <15 mmHg) and the high-pressure group (bIOP ≥15 mmHg). For normally distributed data, intergroup comparisons were conducted using independent samples t-tests; otherwise, Mann-Whitney U tests were applied. Categorical variables were analyzed using Pearson’s Chi-squared tests or Fisher’s exact tests when appropriate. Statistical significance was defined as *p* < 0.05.

## 3 Result

A total of 48 corneal stromal lenticule specimens from 26 young myopic adults were included in the analysis. Among them, 22 subjects contributed bilateral data and four contributed unilateral data. The lenticules were stratified into two groups according to bIOP: bIOP <15 mmHg (N = 15) and bIOP ≥15 mmHg (N = 33). The primary analysis treated each lenticule as an independent observation. A sensitivity analysis using only one randomly selected eye per subject (N = 26) yielded consistent results, suggesting that inter-eye correlation did not substantially affect the conclusions. There were no statistically significant differences in baseline characteristics such as age, gender distribution, pre-operative central corneal thickness (CCT), BGF, and parameters related to the cap, stromal lenticule, and remaining stroma bed thickness from the SMILE procedure between the two groups (all *p* > 0.05; Table 2).

**TABLE 2:** Demographic and clinical data of the involved eyes (48 eyes from 26 patients).

Characteristic	Overall N = 48^1^	bIOP <15 mmHg N = 15^1^	bIOP ≥15 mmHg N = 33^1^	*p*-value^2^
Gender				0.7
Female	35 (73%)	12 (80%)	23 (70%)	
Male	13 (27%)	3 (20%)	10 (30%)	
Age, year	26 (7)	25 (6)	27 (7)	0.5
Eye				0.3
OD	25 (52%)	6 (40%)	19 (58%)	
OS	23 (48%)	9 (60%)	14 (42%)	
SE, diopter	−5.07 (1.33)	−5.19 (0.90)	−5.02 (1.49)	0.7
CR, mm	43.24 (1.47)	42.70 (1.24)	43.48 (1.51)	0.1
IOP, mmHg	16.59 (2.36)	14.30 (0.84)	17.64 (2.08)	<0.001
bIOP, mmHg	15.98 (2.08)	13.76 (0.72)	16.99 (1.66)	<0.001
CCT, μm	561 (29)	567 (25)	558 (30)	0.3
ARTh	687 (181)	632 (135)	712 (195)	0.2
SPA1	107 (13)	98 (8)	111 (13)	<0.001
CBI	0.017 (0.056)	0.031 (0.087)	0.011 (0.034)	0.3
TBI	0.19 (0.31)	0.31 (0.41)	0.07 (0.13)	0.2
SSI	0.97 (0.13)	0.91 (0.10)	1.00 (0.13)	0.03
BGF	0.14 (0.10)	0.12 (0.11)	0.15 (0.10)	0.4
Cap diameter, mm	7.47 (0.18)	7.49 (0.21)	7.46 (0.16)	0.7
Cap thickness, μm				0.2
120	11 (23%)	2 (13%)	9 (27%)	
125	34 (71%)	11 (73%)	23 (70%)	
130	3 (6.3%)	2 (13%)	1 (3.0%)	
Lenticule diameter, mm	6.59 (0.18)	6.61 (0.22)	6.58 (0.16)	0.6
Lenticule overlap, mm				0.7
0	10 (21%)	4 (27%)	6 (18%)	
0.1	38 (79%)	11 (73%)	27 (82%)	
Lenticule thickness, μm	108 (24)	108 (18)	108 (26)	>0.9
Lenticule basethickness, μm				
10	48 (100%)	15 (100%)	33 (100%)	

^1^n (%); Mean (SD).

^2^Fisher’s exact test; One-way analysis of means; Pearson’s Chi-squared test.

Abbreviation: OD, right eye (Oculus Dexter); OS, left eye (Oculus Sinister); SE, spherical equivalent; IOP, intraocular pressure; bIOP, biomechanically corrected IOP; CCT, central corneal thickness; ARTh, Ambrósio’s Relational Thickness horizontal direction; SPA1, Stiffness Parameter A1; CBI, corvis biomechanical index; TBI, tomographic and biomechanical index; BGF, biomechanical glaucoma factor; SSI, Stress-Strain Index; CR, corneal curvature radius.

**FIGURE 1:**
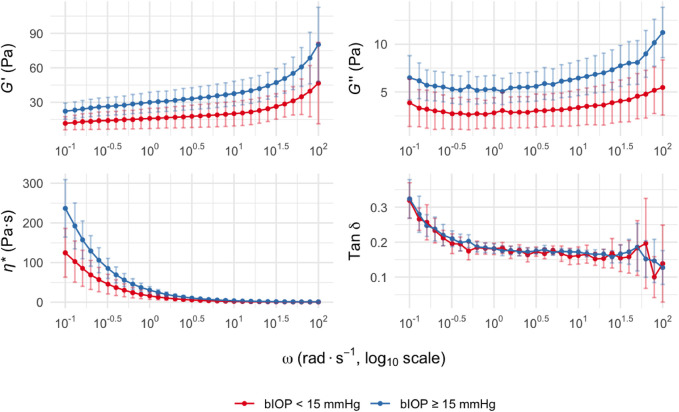
Viscoelastic parameters of corneal stromal lenticules at different oscillatory frequencies. *G′*, *G″*, η*, and tan *δ* are plotted versus *ω*. Data are presented as mean with 95% confidence intervals for two groups stratified by bIOP: bIOP <15 mmHg (red curves) and bIOP ≥15 mmHg (blue curves). Data are presented as mean values with 95% confidence intervals. Abbreviation: *G′*, Storage modulus; *G″*, loss modulus; η*, complex viscosity; tan *δ*, loss tangent; *ω*, angular frequency; bIOP, biomechanically corrected intraocular pressure.

The viscoelastic properties of the lenticules were assessed across a range of oscillatory angular frequencies. The *G′*, *G″*, η*, and tan *δ* all exhibited frequency-dependent changes in both groups (Figure 1).

Across the measured frequency range (0.1–100 rad s^-1^), the bIOP ≥15 mmHg group consistently showed higher mean values of *G′* than the bIOP <15 mmHg group, indicating increased corneal stiffness. This trend was also observed for the *G″*, suggesting enhanced viscous damping properties in the higher bIOP group. The differences between groups were statistically significant at *ω* of 10^0^–10^1.5^ rad s^-1^ and above (*p* < 0.05). Similarly, the η* was elevated in the bIOP ≥15 mmHg group compared to the lower bIOP group throughout the tested frequency range. The tan *δ* values, reflecting the viscoelastic balance between *G′* and *G″*, displayed no significant intergroup differences across most *ω*.

**FIGURE 2:**
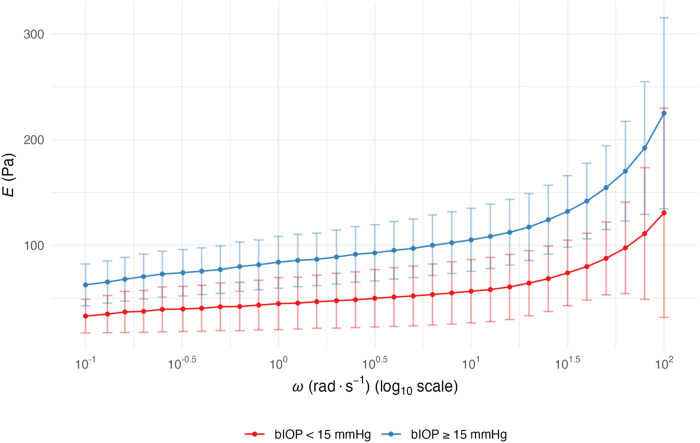
*ω*-dependent *E* of corneal stromal lenticules stratified by bIOP. Data are presented as mean with 95% confidence intervals for two groups stratified by bIOP: bIOP <15 mmHg (red curves) and bIOP ≥15 mmHg (blue curves). Data are presented as mean values with 95% confidence intervals. Abbreviation: *ω*, angular frequency; *E*, elastic modulus; bIOP, biomechanically corrected intraocular pressure.

The elastic modulus *E* was calculated from the storage modulus *G*′ using *E = 2G′(1+ν)* with Poisson’s ratio *ν* = 0.4. Across the tested *ω*, range (0.1–100 rad s^-1^, log_10_ scale), *E* displayed the expected frequency dependence, rising steadily toward the high-*ω* end in both groups (Figure 2). Eyes with bIOP ≥15 mmHg exhibited significantly higher mean *E* values than those with bIOP <15 mmHg throughout the 10^0^–10^1.5^ rad s^-1^ spectrum (*p* < 0.05). The between-group separation became most conspicuous at ≥ 1 rad s^-1^, indicating that elevated IOP is accompanied by a stiffer corneal matrix, especially under rapid, small-amplitude deformation. The curves nearly converged at the lowest *ω* but diverged markedly above 10 rad s^-1^. Linear mixed-model analysis indicated comparable *ω*-response slopes between groups, implying similar viscoelastic dispersion but a higher overall stiffness offset in the high-bIOP eyes.

## 4 Discussion

This study establishes a clear association between bIOP and corneal viscoelastic behavior. Using small-strain, strain-controlled torsional shear rheometry, we quantified the frequency-dependent response of stromal lenticules harvested during SMILE in a deformation mode that closely mirrors the subtle rotational micro-strains encountered *in vivo*. This bench-scale characterization directly addresses the physiological regime where the cornea operates under 1% strains as an approximately linear viscoelastic solid.

Across the examined frequency spectrum, *G′*, *G″*, and the derived *E* rose toward the high-frequency end, as expected for a viscoelastic solid. Eyes with higher bIOP exhibited a uniform upward shift in these moduli at every tested frequency, whereas the rate at which each modulus changed with frequency remained essentially constant across pressure groups. In practical terms, elevated bIOP increases the baseline stiffness of the corneal matrix without measurably altering the underlying time-dependent relaxation mechanisms that govern energy storage and dissipation. Clinically, this aligns with reports that corneas in normal-tension glaucoma are more deformable, whereas those in high-tension glaucoma and ocular hypertension are comparatively less deformable (Liu et al., 2023). The present work provides direct torsional-shear evidence consistent with that trend and supports the view that healthy corneas are stabilized against large deformations by stress-stiffening mechanisms (McMonnies and Boneham, 2007). A plausible structural explanation is a modest increase in collagen packing density or cross-link density that stiffens the fibrillar backbone while leaving the molecular relaxation spectrum largely intact. Whether such material changes are a cause or a consequence of higher IOP cannot be resolved in a cross-sectional study; longitudinal designs that integrate *in vivo* imaging with *ex vivo* rheometry are warranted.

These findings also dovetail with organ-scale *in vivo* metrics obtained using the Corvis ST. Pressure-independent indices such as bIOP, together with DCR-derived parameters that link corneal biomechanics to ocular disease—for example, the BGF (Pillunat et al., 2019)—reflect resistance to transient loading under physiological conditions. Considered alongside our *ex vivo* modulus, the data suggest that within the physiological IOP range, higher baseline pressure elevates the cornea’s stiffness “offset” while maintaining the ratio of stored to dissipated energy. Distinguishing an overall stiffness shift from invariant tan *δ* may refine constitutive models and inform early risk stratification in pressure-related ocular disorders, including glaucoma and keratoconus.

Understanding how IOP, eye rubbing, and stromal mechanics interact is particularly important for translational relevance (Ben-Eli et al., 2019). Eye rubbing directly perturbs corneal optics and transiently elevates IOP; experimental application of “light” and “firm” digital forces to eyes with a baseline IOP of 15 mmHg can increase IOP by approximately twofold and fourfold, respectively (McMonnies and Boneham, 2007; McMonnies, 2008). Repetitive mechanical stimulation may also promote corneal thinning and reduce rigidity, potentially increasing the risk of ectasia such as keratoconus (Balasubramanian et al., 2013; Dou et al., 2022). In eyes with elevated bIOP, remodeling of the stromal matrix could further stiffen tissue and enhance resistance to torsional stress (Petroll et al., 2020). However, the torsional stresses and strains produced by vigorous eye rubbing may exceed the magnitudes tested here; future studies should examine whether corneas with different baseline bIOP exhibit differential resilience to acute IOP spikes and whether this modulates susceptibility to keratoconus onset or progression.

The bIOP-stiffness association may inform refractive surgery planning. Lower baseline bIOP (reduced stiffness) could increase post-SMILE ectasia risk with aggressive ablation (Sinha Roy and Shetty, 2017), whereas higher bIOP may expand safety margins for high-myopia corrections. For lenticule transplantation (Ganesh et al., 2014), bIOP-matched donor-recipient pairing could optimize biomechanical integration. Pre-operative bIOP assessment may thus refine patient selection for precision refractive surgery.

This study has several limitations. Lenticules were sourced from a narrow age range of myopic SMILE patients, which may not extrapolate to pediatric or elderly populations with age-related collagen differences (Elsheikh et al., 2007; Whitford et al., 2015). Tissue hydration was controlled via BSS immersion at 37 °C, but *ex vivo* conditions may differ from *in vivo* endothelial regulation, and potential swelling artifacts could slightly overestimate compliance. The tested strain amplitude (1%) and frequency range (0.1–100 rad s^-1^) interrogated the linear viscoelastic regime but may not capture strain-rate-dependent stiffening or nonlinear responses under rapid transient deformations (Whitford et al., 2018). An isotropic material assumption was applied despite documented stromal anisotropy (Meek and Knupp, 2015). Additionally, rheometry cannot resolve depth-dependent heterogeneity across stromal layers. Future work should integrate Brillouin optical microscopy for depth-resolved stiffness (Scarcelli et al., 2012), optical coherence elastography for strain visualization (Wang and Larin, 2015), second-harmonic generation for collagen architecture, and finite element modeling with patient-specific geometry and anisotropic constitutive laws to simulate pathological loading scenarios such as acute IOP spikes or post-surgical remodeling (Simonini and Pandolfi, 2015). This multiscale framework would enable translation of bIOP-modulus relationships into individualized risk prediction tools for keratoconus, glaucoma, or post-refractive ectasia.

In summary, strain-controlled torsional-shear rheometry reveals that higher physiological IOP is mirrored by a consistent upward shift in corneal stiffness, while the viscoelastic loss factor remains unaltered. This separation of a stiffness offset from invariant the viscoelastic loss factor offering a path to refine constitutive models and to develop biomechanical markers for early risk stratification in pressure-related ocular diseases.

## Data Availability

The raw data supporting the conclusions of this article will be made available by the authors, without undue reservation.
